# *MX2* and *IRF7* are Associated With Disease Activity and Renal Involvement in Systemic Lupus Erythematosus: An Exploratory Study

**DOI:** 10.1155/jimr/8603997

**Published:** 2026-07-26

**Authors:** FengQi Zhang, YiChen Huang, Hang Liu, YiYang Zhang, ZhiYu Li, ZhiJun Xie, Jing Sun

**Affiliations:** ^1^ School of Basic Medical Sciences, Zhejiang Chinese Medical University, Hangzhou, China, zcmu.edu.cn; ^2^ Innovative Institute of Chinese Medicine and Pharmacy, Shandong University of Traditional Chinese Medicine, Jinan 250355, China, sdutcm.edu.cn; ^3^ Department of Internal Medicine, Yuyao Hospital of Traditional Chinese Medicine, Yuyao, Zhejiang, China; ^4^ The First Affiliated Hospital of Zhejiang Chinese Medical University (Zhejiang Provincial Hospital of Chinese Medicine), Hangzhou, China, zjhtcm.com; ^5^ China and Zhejiang University of Traditional Chinese Medicine Jinhua Research Institute, Hangzhou, China; ^6^ The Second School of Clinical Medicine, Zhejiang Chinese Medical University, Hangzhou, China, zcmu.edu.cn

**Keywords:** bioinformatics analysis, machine learning, SLEDAI, systemic lupus erythematosus, type I interferon

## Abstract

**Background:**

Systemic lupus erythematosus (SLE) has multiple phenotypes, one of which is lupus nephritis (LN), a very serious complication with a high mortality rate. Patients with LN may require different treatment regimens than patients with SLE, as there are significant differences between SLE and LN pathogenesis; however, to date, no biomarkers specific for LN have been characterized. Identification of immunological markers of LN could therefore facilitate more individualized treatment strategies.

**Materials and Methods:**

Using public datasets (GSE121239), we screened patients based on SELENA‐SLEDAI scores into mild (SLELA), moderate‐to‐severe (SLEHA), and healthy control (HC) groups. Bioinformatics analyses identified differentially expressed genes (DEGs), which were analyzed for functional pathway enrichment and immune cell infiltration. We validated these findings in an independent clinical cohort of 40 SLE patients (20 with LN and 20 without LN) and 20 HC individuals. LN diagnosis was confirmed by renal biopsy or clinical criteria. We assessed disease activity using clinical indicators and measured the expression of *MX2*, *IRF7*, and *TRIM69* in peripheral blood mononuclear cells (PBMCs) via real‐time quantitative PCR (*n* = 40). Serum interferon‐α (IFN‐α) levels were measured by ELISA, and protein expression in kidney tissue was detected by immunofluorescence (IF).

**Results:**

We observed differential expression of 32 disease activity‐related genes in SLE. Univariate logistic regression and ROC curve analysis indicated that *MX2* (AUC = 0.95, 95% CI: 0.92–0.98), *TRIM69* (AUC = 0.93, 95% CI: 0.89–0.96), and *IRF7* (AUC = 0.91, 95% CI: 0.87–0.95) were associated with SLE diagnosis. Gene ontology functional enrichment analysis suggested that the type I IFN pathway is aberrantly activated in SLE. Disease activity‐related genes such as *MX2*, *IRF7*, and *IFIT3* are functionally enriched in the type I IFN pathway, and abnormal infiltration of neutrophils is involved in SLE pathogenesis. Disease activity‐related genes such as *MX2* and *IRF7* were positively correlated with immune cell infiltration, including neutrophils and memory B cells. RT‐qPCR and ELISA showed significantly higher expression of IFN‐α, *MX2*, and *IRF7* in LN patients than in SLE patients. IF assays showed that IFN‐α, *MX2*, and *IRF7* were expressed at significantly higher levels in the kidneys of LN patients than in SLE patients.

**Conclusions:**

Our study identifies MX2 and IRF7 as candidate biomarkers associated with high systemic disease activity in SLE. In our validation cohort, their expression was significantly elevated in patients with LN, suggesting they may serve as potential noninvasive indicators warranting further longitudinal investigation for LN risk assessment.

## 1. Introduction

Lupus nephritis (LN) is a serious renal complication triggered by systemic lupus erythematosus (SLE), developing in ~40%–60% of patients early in the disease course [1]. The pathogenesis of LN is multifactorial and heterogeneous [2]. In SLE, immune complex formation and impaired clearance of cellular debris, including apoptotic material and neutrophil extracellular traps (NETs) enhance nucleic acid sensing and downstream lymphocyte activation, alongside dysregulated type I interferon (IFN) signaling, together driving kidney inflammation and injury [3]. Clinically, LN diagnosis and risk stratification rely on integrating laboratory findings with renal histopathology, often requiring an invasive kidney biopsy. Therefore, a simple, noninvasive method to assess the LN risk and monitor renal involvement is urgently needed.

Aberrant production of antiviral IFN and increased expression of IFN‐regulated genes in the blood and tissue are well‐established drivers of SLE and LN pathogenesis [4]. In particular, type I IFNs act as immune adjuvants and stimulate T and B cells and monocytes [5], which together mediate the production of “high IFN characteristics” in target organ tissues such as the kidney, causing vascular tissue damage [6] and initiating SLE and organ damage. Current therapies targeting the type‐I IFN pathway show promising potential, underscoring the possibility that IFN signaling may serve as a more informative biomarker of overall disease severity than conventional disease activity assessments [7]. However, although a type I IFN signature is a well‐established feature of SLE, defining the full transcriptomic signature in routine clinical settings remains complex [8]. Therefore, a streamlined panel comprising a small set of representative genes may be more practical for clinical diagnosis and monitoring. Among type I IFN‐induced genes, *MX2* and *IRF7* are expressed at high levels in patients with SLE and have been linked in other studies to pathways that could promote neutrophil infiltration, such as NOD‐like receptor signaling [9, 10]. Yet, IFN activity is typically captured using broad, heterogeneous gene modules that are difficult to standardize in routine practice and may not distinguish kidney involvement from systemic inflammation [11]. This highlights a key unmet need for defining LN‐specific gene signatures that reliably identify and stratify renal involvement. Accordingly, the objective of this study was to derive a streamlined panel of surrogate IFN‐related genes and determine whether these markers—particularly MX2 and IRF7—form a reproducible transcriptomic signature that is specifically associated with LN in a well‐characterized clinical cohort [12, 13].

Recent advances in bioinformatic analysis techniques have greatly expanded our ability to dissect the molecular mechanisms that drive disease onset and progression [14]. In this study, we integrated previously published peripheral whole‐blood microarray data from patients with SLE and healthy controls (HCs) with a comprehensive gene expression database to systematically identify disease activity‐associated genes using an unbiased bioinformatics pipeline. We then performed functional enrichment analyses, characterized immune cell infiltration patterns, and prioritized candidate genes linked to key disease activity pathways. Finally, we validated the expression of these candidates in an independent clinical cohort of patients with SLE, including individuals with LN. Together, our results nominate circulating biomarkers associated with renal involvement and support their potential utility for noninvasive LN risk assessment [15].

## 2. Materials and Methods

### 2.1. Data Collection and Processing

To gather relevant data, we selected the Gene Expression Omnibus (GEO) in the NCBI database (https://www.ncbi.nlm.nih.gov/geo) and searched using the keyword “SLE” and the following screening criteria: (1) the data contained gene sequencing expression profiles belonging to *Homo sapiens*, (2) the data were derived from whole blood processed, (3) the datasets contained both patient and control groups, (4) raw data files and annotation files were accessible and downloadable, (5) raw data files contained the SELENA‐SLEDAI disease activity score, and (6) the files contained sequencing data at baseline and follow‐up. The use of the public GEO dataset complied with the original informed consent and data‐sharing policies of the repository. The dataset GSE121239 was downloaded, the raw data were imported using R (version 4.0.1), clinical information was read using the “oligo” package, and the RMA method was applied for unified matrix normalization. Patients were grouped according to the SELENA‐SLEDAI disease activity score, following the classification criteria: no disease activity (0–4), mild activity (5–9), moderate activity (10–14), and severe activity (≥15).

### 2.2. Differential Gene Expression Analysis

Genes identified as differentially expressed between mild disease activity, moderate‐to‐severe disease activity, and healthy were assessed using the “limma” package, and *p* values were calculated using empirical Bayes methods. The following cutoff criteria were used to identify differentially expressed genes (DEGs): |log_2_ (fold change)| ≥1 and *p* < 0.05. Heat maps illustrating the expression patterns of DEGs were drawn with the “ggplot2” package.

### 2.3. Weighted Gene Coexpression Network Analysis (WGCNA)

WGCNA can partition gene coexpression networks into multiple highly correlated signature modules and associate modules with clinical phenotypes, thereby identifying biomarkers involved in biological processes. First, a coexpression network was constructed using the “WGCNA” package to search for biologically or clinically meaningful modules and genes in the mild disease activity and moderate to‐severe disease activity groups. Genes were ranked according to gene expression, and genes with the top 5000 expressions were selected for the next step of analysis. Second, the function “pickSoftThreshold” was used to select the appropriate soft threshold (soft thresholding power) to calculate network adjacency and construct the topological overlap matrix. We used hierarchical clustering and dynamic tree segmentation functions to identify the modules. Module–clinical trait relationship heat maps were produced, and the most relevant module with SLE disease activity was selected as the key module associated with SLE activity. We used gene significance (GS) and module membership (MM) to correlate modules with clinical features such as moderate‐to‐high disease activity. In agreement with related studies, |MM| > 0.7 and |GS| > 0.15 were used as screening criteria for module hub genes.

### 2.4. Protein–Protein Interaction (PPI) Network Generation and Gene Screening

Using Venny (https://bioinfogp.cnb.csic.es/tools/venny/index.html), a Wayne diagram was drawn to identify the intersection of DEGs between medium and high disease activity and module hub genes, and SLE marker genes and activity‐related genes were selected. Intersecting genes were imported into the string database (http://string-db.org/), and a PPI of DEGs was constructed and imported into Cytoscape to form a network core map. The top 10 core genes were then obtained using the DMNC algorithm.

### 2.5. Univariate Logistic Regression and ROC Analysis

Univariate logistic regression models were constructed in R to assess the association between candidate gene expression and SLE diagnosis. Odds ratios (ORs) and 95% confidence intervals (CIs) were calculated. Forest plots were generated using the “forestplot” package. To evaluate diagnostic performance, receiver operating characteristic (ROC) curve analysis was performed using the “pROC” package, with the area under the curve (AUC) calculated for MX2, IRF7, and TRIM69.

### 2.6. GO Analysis

The selected DEGs were included in the GO enrichment analysis, and GO entries significantly enriched in DEGs were screened using a threshold of “*p* value <0.05” to draw the corresponding bubble diagram and pathway relationship diagram. GO functional enrichment for each module was analyzed using the “WGCNA” package, and functional enrichment analysis results of selected modules linked to clinical phenotypes were confirmed and visualized.

### 2.7. Immune Cell Infiltration Analysis

To perform immune cell infiltration analysis, we used CIBERSORT, one of the most commonly used algorithms to calculate immune cell infiltration. Using the gene expression matrix, the abundances of 22 immune cell subsets were deduced using the LM22 signature matrix, which is validated for whole blood deconvolution, with 1000 permutations,;intersection genes were analyzed for association with immune cells, and the selected results were visualized.

### 2.8. Blood Specimen Collection From Patients With SLE

SLE patients (*N* = 40; aged 18–65 years), including newly diagnosed outpatients and inpatients, as well as those deteriorating to the point of requiring hospitalization, were recruited. Peripheral blood samples were collected and compared with samples from 20 healthy volunteers recruited from the Department of Rheumatology of the First Affiliated Hospital and the Second Affiliated Hospital of Zhejiang Chinese Medical University.

To assess the disease activity, we used the SELENA‐SLEDAI score. All SLE and LN patients in the validation cohort were enrolled at the time of initial diagnosis. To minimize drug‐related confounding by type I IFN signature, only newly diagnosed patients were included. The following exclusion criteria were used: patients with SLE that were treated for more than 3 months, patients currently taking targeted agents such as rituximab, patients who lacked legal capacity, systemic glucocorticoids for SLE, antimalarials such as hydroxychloroquine, conventional or biologic immunosuppressive agents (e.g., mycophenolate mofetil, cyclophosphamide, azathioprine, methotrexate and, rituximab), patients who could not understand the study risks and follow‐up requirements, patients with a history of drug abuse, and patients with complex medical conditions such as malignancies or blood disorders. All included persons were divided into SLE and LN according to the SELENA‐SLEDAI score. Meanwhile, clinical information such as clinical symptoms and laboratory indicators was also collected. To minimize the confounding effects of medication on IFN signatures, all enrolled SLE and LN patients were newly diagnosed and treatment‐naïve at the time of sampling. Patients who had received systemic glucocorticoids, hydroxychloroquine, or immunosuppressants prior to the sample collection were excluded. All participants provided informed consent according to ethical standards, all procedures involving human participants were performed in accordance with the Declaration of Helsinki, and all experimental protocols were approved by the ethics committee (ethics number: 2022 research number: 086‐01). Blood samples (5 mL) were collected from patients and healthy individuals and anticoagulated with EDTA. PBMCs were isolated using Ficoll‐Paque density gradient separation and cryopreserved until use. A flow diagram detailing patient recruitment and group allocation is provided in Figure S1. All biological samples were de‐identified and stored securely at −80°C. Patient clinical data were anonymized and strictly managed to ensure privacy and comply with data protection regulations.

### 2.9. Real‐Time Quantitative PCR Assays

The RNAiso Plus reagent (TAKARA, 9108) was used to extract total RNA from PBMCs, and cDNA was synthesized using an iScript cDNA synthesis kit (Bio‐Rad, 1708891). Relative quantitation of target genes was performed using a Taq Pro Universal SYBR Green qPCR kit (Accurate Biology, AG11701), and *GAPDH* served as the housekeeping gene. PCR was performed using a LightCycler 96 (Roche, Basel, Switzerland) and the following primers: *MX2*: forward 5′‐CAGAGGCAGCGGAATCGTAA‐3′ and reverse 5′‐TGAAGCTCTAGCTCGGTGTTC‐3′; I*RF7*: forward 5′‐TGGCAGACCATCTGCTGACA‐3′ and reverse 5′‐GCCTTGGTTGGGACTGGAT‐3′; *TRIM69*: forward 5′‐TGGCAGCAGCGTCGTCATAG‐3′ and reverse 5′‐GTTGTTCCGTCTTTGCCTGAATGC‐3′; *GAPDH*: forward 5′‐CACCCACTCCTCCACCTTTGAC‐3′ and reverse 5′‐GTCCACCACCCTGTTGCTGTAG‐3′.

### 2.10. Enzyme‐Linked Immunosorbent Assay and Blood Biochemical Test Indicators

Levels of IFN‐α (CSB‐E08636h) in serum acquired were determined using an ELISA kit (Human, China), and absorbance was assessed using a microplate reader (TECAN, INFINITE 200 PRO, Austria).

### 2.11. Immunoblotting

PBMC cells were subjected to lysis on ice in RIPA buffer supplemented with phosphatase and protease inhibitors to extract the total protein. Following centrifugation at 4°C, 12,000 rpm for 10 min, the total protein concentration was measured using the Pierce BCA kit. An equal protein concentration from each sample was then boiled for 10 min in 5x loading buffer and separated by SDS‐PAGE (Epizyme). Proteins were transferred to the PVDF membrane, which was subjected to blocking in 5% skimmed milk/PBS, and then incubated with primary antibodies overnight at 4°C. The following day, after washing three times, secondary antibodies were applied to the membrane for 1 h. A ChemiDoc imaging system (Bio‐Rad) was used to visualize protein bands. The primary antibodies used were raised against IFN‐α, MX2, IRF7, TRIM69, and GAPDH. GAPDH served as the loading control for normalization.

### 2.12. Renal Multiplex Immunofluorescence (IF) Staining

Human paraffin‐embedded kidney tissue sections, which were obtained as part of routine clinical diagnostic procedures rather than research‐specific interventions, were prepared for IF by staining with primary antibodies, including anti‐IFN‐α (Thermo Fisher, PA5‐119649), anti‐MX2 (Thermo Fisher, PA5‐102005), anti‐IRF7 (Thermo Fisher, MA5‐41165), and anti‐TRIM69 (Thermo Fisher, PA5‐117032), and incubating overnight at 4°C. Tissues were then incubated for 60 min at room temperature with species‐specific secondary antibody (horseradish peroxidase), washed, treated with DAPI to stain the nucleus, and mounted with antifluorescence quenching mounting medium. Sections were then imaged with a fluorescence microscope.

### 2.13. Statistical Analysis

Statistical analyses were performed using R (version 4.0.1) and GraphPad Prism. Continuous variables are presented as mean ± standard deviation (SD) for normally distributed data or median (interquartile range, IQR) for nonnormally distributed data. Differences between two groups were analyzed using the Student’s *t*‐test or Mann–Whitney *U* test. Comparisons among three or more groups were performed using one‐way ANOVA or the Kruskal–Wallis test. Correlations were assessed using the Spearman’s rank correlation coefficient. *p* < 0.05 was considered statistically significant.

## 3. Results

### 3.1. Differential Analysis

**Figure 1:**
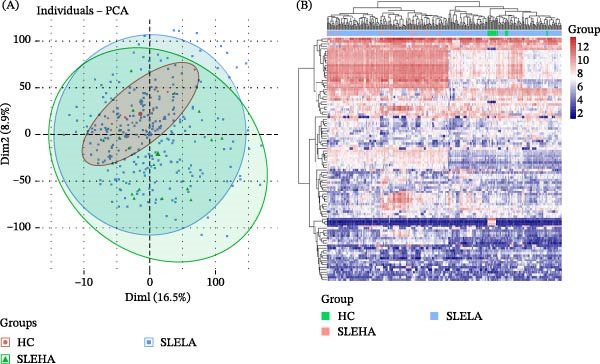
Differential gene expression analysis between SLEHA, SLELA, and HC. (A) Principal component analysis. The *x*‐axis (Dim1) and *y*‐axis (Dim2) represent the top two principal components, accounting for 16.5% and 8.9% of the total variance. (B) Heat map showing DEGs in each sample from SLEHA and SLELA patients and HC individuals. Dim, dimension; PCA, principal component analysis.

Using the data available within GSE121239, DEGs were identified between individuals with mild disease activity (SLELA), those with moderate to severe disease activity (SLEHA), and HCs. Visualization of the distribution of gene expression patterns reflected the heterogeneity between different activity levels in individuals with SLE and HC (Figure 1A), and gene expression patterns clustered based on disease status (Figure 1B), providing a basis for the analysis of signature genes representing disease activity in SLE.

### 3.2. Weighted Gene Coexpression Network (WGCNA) Analysis

**Figure 2:**
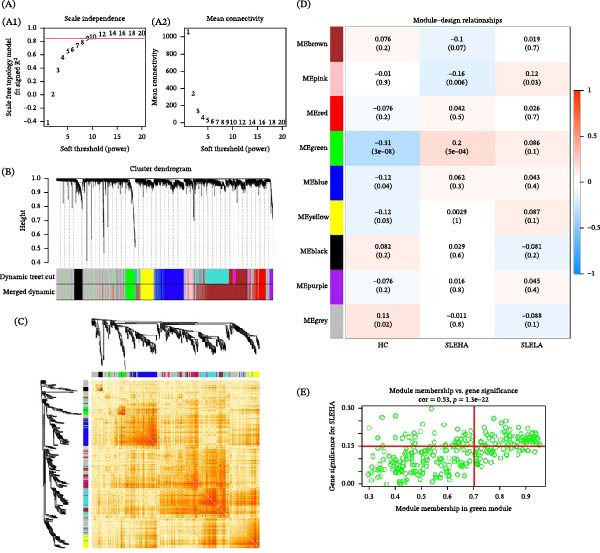
WGCNA. (A) Selection of the soft‐thresholding power (*β* = 9) to achieve a scale‐free topology network (A1) and mean connectivity (A2). (B) Similarity drawing hierarchical clustering tree. (C) Heat map of intergenic similarity within different modules. A darker color indicates higher topological overlap. (D) Module–clinical trait relationship heat map divided into SLEHA, SLELA, and HC as clinical traits, in which the green module (Megreen) was positively correlated with SLEHA (*r* = 0.2, *p* = 5e − 04). (E) Scatter plots of GS and MM for green module genes (Megreen), with |MM| >0.7 and |GS| > 0.15 used as screening criteria for module‐hub genes.

Following WGCNA, the dataset was divided into different gene modules according to disease activity to obtain gene networks with moderate to high activity, and eligible module–clinical trait genes were screened. A suitable soft threshold of 9 was determined to construct the network, and gene expression values were converted into expression similarity between genes to construct a similarity matrix (Figure 2A). According to the similarity degree of the above‐described genes, a hierarchical clustering tree was drawn (Figure 2B), with similarity values ranging from 0 to 1, with 1 indicating the highest similarity between genes. The hierarchical clustering tree converged genes with similar expression patterns into clusters to divide different coexpression modules and symbolize different modules with different colors. Intergenic similarity within different modules was assessed, and further analysis merged similar modules and finally retained nine modules (Figure 2C). A heat map of module–clinical trait relationships was drawn using disease activity as the clinical phenotype classification criteria (Figure 2D), in which the green module (Megreen) was positively correlated with moderate to severe disease activity in SLE, and the correlation coefficient was the highest (*p* < 0.05); |MM| >0.7 and |GS| >0.15 were used as screening criteria for module‐hub genes to screen module–clinical trait genes in green modules that met the screening criteria (Figure 2E).

### 3.3. Construction of PPIs and Gene Screening

**Figure 3:**
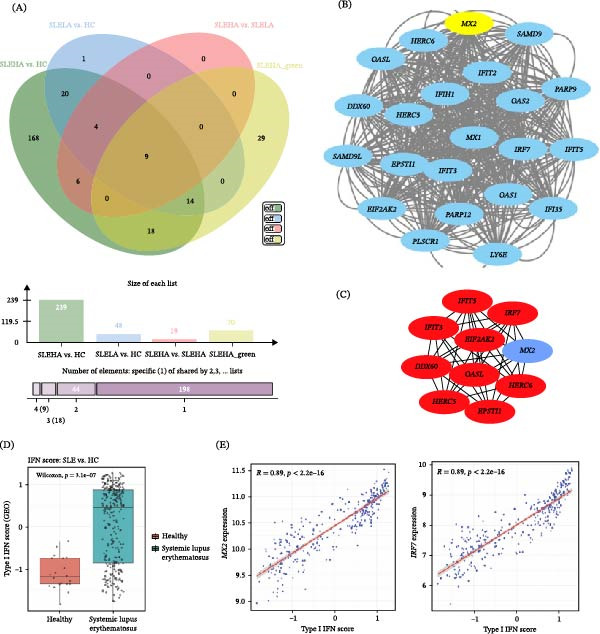
PPI network construction and gene screening. (A) Wayne diagram of the intersection of DEGs and module–clinical trait genes derived from the WGCNA to derive marker genes and disease activity‐related genes in SLE. (B) Clusters of intersection genes by MCODE analysis. (C) Clusters of genes identified by cytohubba‐DMNC analysis. (D) Boxplot showing the type I IFN score is significantly elevated in SLE patients compared to healthy controls (Wilcoxon test, *p* < 0.001). (E) Scatter plots showing a strong positive correlation between the type I IFN score and the expression of MX2 (*R* = 0.89, *p* < 0.001) and IRF7 (*R* = 0.89, *p* < 0.001), supporting their utility as surrogate biomarkers.

The DEGs and module–clinical trait genes derived from the WGCNA were intersected and visualized by the Wayne plot. Forty‐one DEGs between SLEHA and HC and intersection genes between module–clinical trait genes were identified (Figure 3A). Nine of these genes were also differentially expressed between SLELA and HC and between SLEHA and SLELA, which were regarded as SLE marker genes, including *RSAD2*, *IFIT1*, *ISG15*, *IFI44*, *IFI44L*, *OAS3*, *IFI27*, *CMPK2*, and *SIGLEC1*. After removing the marker genes, the remaining 32 intersection genes were regarded as SLE disease activity‐related genes, including *MX2*, *IRF7*, *IFIT3*, *IFIT5*, *EIF2AK2*, *DDX60*, *HERC5*, *HERC6*, *OASL*, and *EPSTI1*, among others.

PPIs of genes related to disease activity were constructed using string databases, and PPI networks were visualized using Cytoscape. Figure 3B shows the clustering of genes derived from the MCODE plug‐in analysis, and Figure 3C shows the clustering of genes derived from the cytohubba‐DMNC plug‐in analysis, which contain interacting genes such as *MX2*, *IRF7*, *IFIT5*, *IFIT3*, *EIF2AK2*, *DDX60*, *HERC5*, *HERC6*, *OASL*, and *EPSTI1*.

We calculated a composite type‐I IFN gene score in the discovery dataset (GSE121239). As expected, the IFN score was significantly higher in SLE patients compared to HCs (*p* < 0.001, Figure 3D). Importantly, correlation analysis revealed that the IFN score was strongly positively correlated with the expression of our identified hub genes, MX2 (*R* = 0.89, *p* < 0.001) and IRF7 (*R* = 0.89, *p* < 0.001) (Figure 3E). These results validate that MX2 and IRF7 are robust representatives of the broader type‐I IFN signaling pathway. Consequently, in the subsequent clinical validation cohort, MX2 and IRF7 were utilized as surrogate biomarkers for IFN activity.

### 3.4. Univariate Logistic Regression Analysis and ROC Analysis

**Figure 4:**
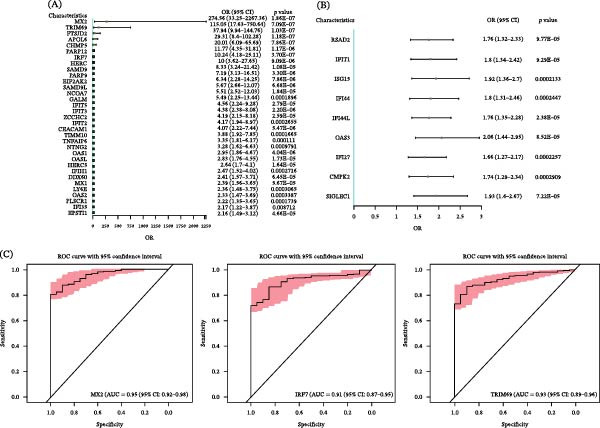
Univariate logistic regression analysis. (A) Univariate logistic regression analysis, representing risk rates for SLE diagnosis for genes associated with disease activity. (B) Risk rates of SLE diagnosis for SLE marker genes. (C) ROC curve analysis for SLE diagnosis in the discovery cohort. AUC values for MX2 (0.95, 95% CI: 0.92–0.98), IRF7 (0.91, 95% CI: 0.87–0.95), and TRIM69 (0.93, 95% CI: 0.89–0.96) demonstrate high discriminative performance.

*MX2* was identified as the gene that was most significantly associated with SLE (OR = 274.56, 95% CI = 33.25–2267.36; *p* < 0.05; Figure 4A), followed by *TRIM69* (OR = 115.05, 95% CI = 17.63–750.64; *p* < 0.05) and *IRF7* (OR = 10.24, 95% CI = 4.18–25.11; *p* < 0.05). *RSAD2*, *IFIT1*, *ISG15*, *IFI44*, and other SLE marker genes were also significantly associated (*p* < 0.05; Figure 4B), however, the ORs were lower compared to MX2 and other disease activity‐related genes, highlighting the distinctive diagnostic potential of MX2 and TRIM69 for SLE.

The ROC curve analysis demonstrated that the key genes identified in our study exhibited strong discriminative ability for distinguishing SLE patients from HCs. As shown in Figure 4C, *MX2* achieved the highest diagnostic performance with an AUC of 0.95 (95% CI: 0.92–0.98), followed by TRIM69 (AUC = 0.93, 95% CI: 0.89–0.96) and IRF7 (AUC = 0.91, 95% CI: 0.87–0.95). The AUC values of all three genes were greater than 0.9, indicating high discriminative potential. These findings suggest that MX2, IRF7, and TRIM69 may serve as potential candidate biomarkers for SLE diagnosis and disease activity assessment.

### 3.5. GO Functional Enrichment Analysis

Biological processes such as “response to virus,” “defense response to virus,” “type I IFN signaling pathway,” “cellular response to type I IFN,” and “response to type I IFN” were determined to be significantly upregulated in SLE (Figure 5A), as assessed by GO functional enrichment analysis. A biological process roadmap of DEGs associated with SLE demonstrated that most genes are involved in “defense response to viruses” and “type I IFN signaling pathway” (Figure 5B). *MX2* and *IRF7*, in particular, are involved in “defense response to viruses,” “type I IFN signaling pathway,” and “response to type I IFN” (Figure 5C). Additionally, genes involved in the type‐I IFN pathway include *MX2*, *IFI27*, *IFIT1*, *IFIT3*, *IRF7*, *ISG15*, *OAS* family, *RASD2*, and *TRIM6*.

Figure 5GO analysis. (A) GO analysis scatter plots of DEGs, including biological process, molecular function, and cellular component analyses. (B) Biological process roadmap for DEGs associated with SLE. (C) Biological processes associated with SLE DEGs. (D) GO functional enrichment analysis of WGCNA modules.

**Figure:**
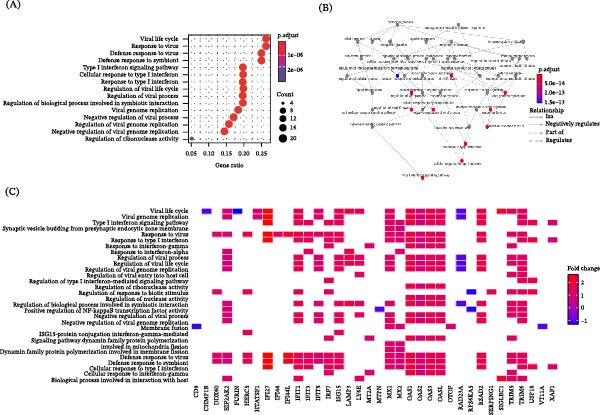

**Figure:**
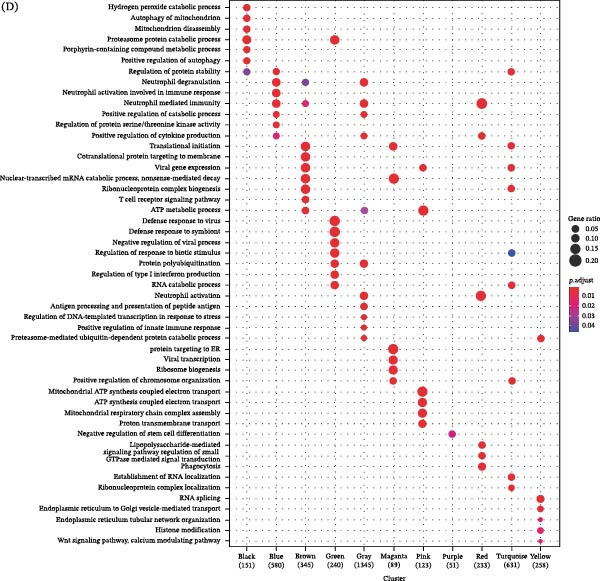

GO functional enrichment analysis of the WGCNA module suggested that green module genes associated with moderate to severe disease activity in SLE are enriched in genes associated with “defense response to virus” and “regulation of type‐I IFN production” (Figure 5D). This is similar to the results discussed above, suggesting that the two analysis methods cluster genes within similar biological processes.

### 3.6. Immune Cell Infiltration Analysis

**Figure 6:**
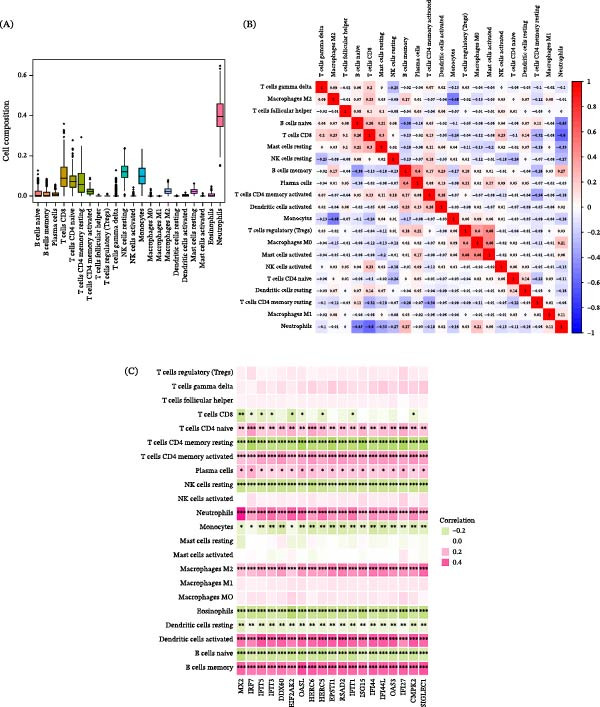
Immune cell infiltration analysis. (A) Immune infiltrate analysis, representing the distribution of immune cell subsets in SLE. (B) Heat map showing the correlations between immune cells. (C) Correlations between SLE disease activity‐related genes, marker genes, and various immune cells. 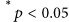, 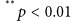, 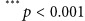.

Using the CIBERSORT algorithm to deconvolve immune cell subsets in whole blood derived from patients with SLE, neutrophils were estimated to have the highest relative abundance, followed by CD8^+^ T cells, naïve CD4^+^ T cells, memory CD4^+^ T cells, natural killer cells, monocytes, B cells, and memory B cells (Figure 6A). This suggests that dysregulation of multiple immune cell populations, particularly neutrophils, may be involved in the pathogenesis of SLE. The estimated proportion of memory B cells showed a strong positive correlation with activated CD4^+^ T cells (Figure 6B).

Correlations were identified between disease activity‐related genes, SLE marker genes, and the predicted abundance of each immune cell type (Figure 6C). *MX2* showed the highest positive correlation with the estimated infiltration levels of neutrophils, memory B cells, M2 macrophages, activated dendritic cells, and activated memory CD4^+^ T cells (*p* < 0.001). The other disease activity‐related genes and SLE marker genes synergize with *MX2* to be positively correlated with the abovementioned immune cells.

### 3.7. Real‐Time Quantitative PCR

**Figure 7:**
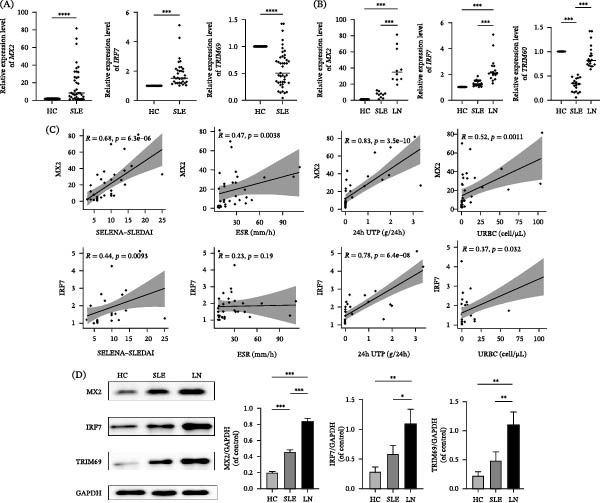
(A) Validation of MX2, IRF7, and TRIM69 expression in the serum of SLE (*n* = 40) and HC patients (*n* = 20). (B) Subgroup analysis of mRNA expression in patients with lupus nephritis (LN, *n* = 20) versus nonrenal SLE (SLE, *n* = 20). (C) Spearman correlation analyses between MX2 and IRF7 expression levels and clinical parameters (SELENA‐SLEDAI score, ESR, 24‐h urinary total protein, urinary red blood cell count, and urinary white blood cell count) in the entire SLE validation cohort (*n* = 40, including 20 LN and 20 nonrenal SLE patients). (D) MX2, IRF7, and TRIM69 protein expression levels were assessed using immunoblotting (*n* = 3). Data are expressed as the mean ± SD. Results indicate a significant improvement in comparison ( ^∗^ *p* < 0.05,  ^∗∗^ *p* < 0.01,  ^∗∗∗^ *p* < 0.001,  ^∗∗∗∗^ *p* < 0.001) group.

No significant differences between age and gender were observed between individuals with SLE and HC (*p* = 0.597 and *p* = 0.999, respectively; Table 1). The expression of *MX2*, *IRF7*, and *TRIM69* was significantly upregulated in the PBMCs of SLE patients compared with that of HC (Figure 7A). However, compared with SLE, the expression of *MX2* and *IRF7* was significantly different in patients with LN, suggesting that MX2 and IRF7 may be more useful for the diagnosis of LN. (Figure 7B). SELENA‐SLEDAI score was positively correlated with the expression of *MX2* (*r* = 0.68, *p* < 0.001) and *IRF7* (*r* = 0.44, *p* = 0.0093), as was ESR (*MX2*: *r* = 0.47, *p* = 0.0038; *IRF7*: *r* = 0.23, *p* = 0.19; Figure 7C). UTP and UWBC were positively correlated with *MX2* and *IRF7* expressions at 24 h, respectively. Among these, 24 h UTP was positively correlated with *MX2* (*r* = 0.83, *p* < 0.001) and *IRF7* expression (*r* = 0.78, *p* < 0.001), as was URBC (*MX2*: *r* = 0.52, *p* = 0.0011; *IRF7*: *r* = 0.37, *p* = 0.032). Immunoblotting of representative samples (*n* = 3 per group) showed protein expression patterns consistent with the mRNA findings, with higher expression of MX2, IRF7, and TRIM69 in LN patients compared with that in HC and SLE patients (Figure 7D).

**Table 1:** Clinical manifestations of SLE and LN patients.

Index	Group		*p*‐Value ^∗^
	SLE (*n* = 20)	LN (*n* = 20)	
Age, mean ± SD, years	39.9 ± 14.7	37.5 ± 11.5	0.576
Gender (women), *n* (%)	18 (90)	17 (85)	>0.999
SELENA‐SLEDAI score, median (IQR)	6 (2.75)	12 (5)	**<0.001**
Psychosis, *n* (%)	0(0)	1(5)	>0.999
Arthritis, *n* (%)	11 (55)	8 (40)	0.527
Myositis, *n* (%)	2 (10)	1 (5)	>0.999
Hematuria, *n* (%)	0 (0)	12 (60)	**<0.001**
Proteinuria, *n* (%)	0 (0)	10 (50)	**0.001**
Rash, *n* (%)	6 (30)	5 (25)	>0.999
Alopecia, *n* (%)	7 (35)	4 (20)	0.479
Mucosal ulcers, *n* (%)	1 (5)	0 (0)	>0.999
Pleurisy, *n* (%)	0 (0)	1 (5)	>0.999
Pericarditis, *n* (%)	0 (0)	1 (5)	>0.999
Low complement, *n* (%)	10 (50)	14 (70)	0.333
Fever, *n* (%)	2 (10)	1 (5)	>0.999
Thrombocytopenia, *n* (%)	5 (25)	3 (15)	0.695
Leukopenia, *n* (%)	2 (10)	3 (15)	>0.999
Anti‐Smith, *n* (%)	2 (10)	9 (45)	**0.034**
Anti‐dsDNA, *n* (%)	6 (30)	8 (40)	0.740
ESR, median (IQR), mm/h	14 (18)	24.5 (25)	0.082
C3, mean ± SD, g/L	0.84 ± 0.26	0.62 ± 0.30	**0.020**
C4, mean ± SD, g/L	0.20 ± 0.08	0.13 ± 0.08	**0.013**
24 h UTP, median (IQR), g/24 h	0 (0)	0.92 (1.81)	**<0.001**
Scr, median (IQR), μmol/L	55.25 (16.17)	52.20 (25.15)	0.499
BUN, median (IQR), mmol/L	4.00 (2.00)	4.00 (1.50)	0.072
Systemic glucocorticoids at sampling, *n* (%)	0 (0)	0 (0)	—
Hydroxychloroquine at sampling *n* (%)	0 (0)	0 (0)	—
Immunosuppressants at sampling *n* (%)	0 (0)	0 (0)	—
ISN/RPS class *n* (%)	0 (0)	III: 2 (2) IV: 9 (45) V: 9(45)	—

*Note:* Data are presented as mean ± SD for normally distributed continuous variables, median (IQR) for non‐normally distributed continuous variables, and *n* (%) for categorical variables. Bold text indicates statistical significance (*p* < 0.05). All patients were newly diagnosed at the time of enrollment; thus, the disease duration at sampling was close to zero. Laboratory reference ranges correspond to the standard clinical parameters of our institution. The symbol “—” indicates that statistical comparison was not applicable or was not performed because all patients in both groups had identical values, or because the variable, such as ISN/RPS classification, was applicable only to patients with lupus nephritis. The  ^∗^ indicates statistical significance at *p* < 0.05.

Abbreviations: BUN, blood urea nitrogen; dsDNA, double‐stranded DNA; ESR, erythrocyte sedimentation rate; IQR, interquartile range; Scr, serum creatinine; SD, standard deviation.

### 3.8. Validation of Model Genes Expression in SLE and LN Serum

**Figure 8:**
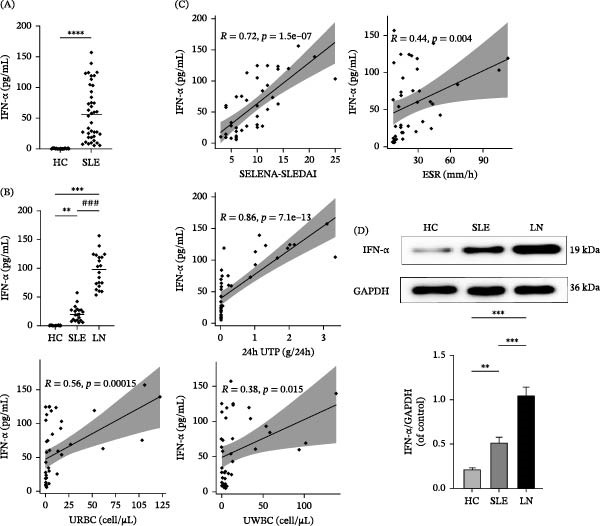
(A) Serum IFN‐α levels measured by ELISA in patients with SLE (*n* = 40) compared to healthy controls (HC, *n* = 20). Data were analyzed using the Mann–Whitney *U* test.  ^∗∗∗∗^ *p* < 0.0001. (B) Subgroup analysis of serum IFN‐α levels in patients with lupus nephritis (LN, *n* = 20) compared to nonrenal SLE patients (SLE, *n* = 20) and HCs (*n* = 20).  ^∗∗^ *p* < 0.01,  ^∗∗∗^ *p* < 0.001 versus HC; ^###^ *p* < 0.001 versus SLE. (C) Correlation analysis of IFN‐α and SELENA‐SLEDAI, ESR, 24 h UTP, URBC, and UWBC. (D) IFN‐α protein expression levels were assessed using immunoblotting (*n* = 3). Data are expressed as the mean ± SD. Results indicate a significant improvement in comparison ( ^∗^ *p* < 0.05,  ^∗∗^ *p* < 0.01,  ^∗∗∗^ *p* < 0.001) group.

Serum IFN‐α levels in SLE patients (*p* < 0.01) and LN (*p* < 0.001) were significantly higher than those observed in HC, and expression levels in LN patients (*p* < 0.001) were significantly higher than those observed in SLE patients (Figure 8A,B).

Spearman correlation analysis showed a positive correlation between SELENA‐SLEDAI score and IFN‐α levels (*r* = 0.72, *p* < 0.001) and ESR and IFN‐α levels (*r* = 0.44, *p* = 0.004). Additionally, UTP and IFN‐α (*r* = 0.86, *p* < 0.001), URBC and IFN‐α (*r* = 0.56, *p* = 0.00015), and UWBC and IFN‐α (*r* = 0.56, *p* = 0.00015) at 24 h were positively correlated (Figure 8C). Western blotting showed significantly higher expression of IFN‐α in LN patients compared with that in HC and SLE patients (Figure 8D).

### 3.9. Effects of IFN‐α Expression and the Expression of IFN‐Related Genes on SLE and LN Patients

**Figure 9:**
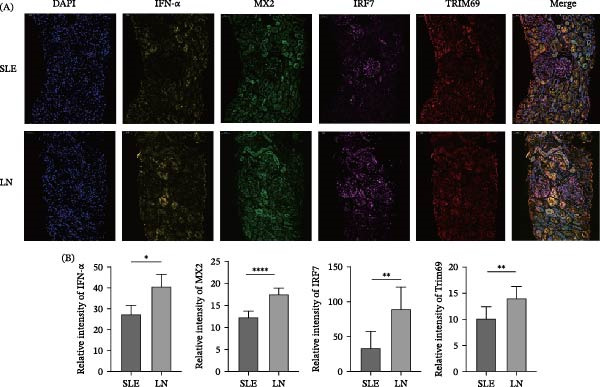
Expression of IFN‐α, *MX2*, *IRF7*, and *TRIM69* in kidney tissue of SLE or LN. (A) Representative multiplex immunofluorescence images showing the expression and localization of IFN‐α (yellow), MX2 (green), IRF7 (magenta), and TRIM69 (red) in human paraffin‐embedded kidney sections from SLE patients (*n* = 5) and LN patients (*n* = 5). Nuclei were counterstained with DAPI (blue). Original magnification, Scale bars = 50 μm. (B) Quantitative analysis of the Mean Fluorescence Intensity (MFI) for each marker measured using ImageJ software. Data are expressed as the mean ± SD from independent microscopic fields. Statistical significance was determined using Student’s *t*‐test.  ^∗^ *p* < 0.05,  ^∗∗^ *p* < 0.01,  ^∗∗∗∗^ *p* < 0.0001.

Next, we investigated the association between the expression of IFN‐related genes and renal pathology injury. We observed that the expression of IFN‐α, *MX2*, and IRF7 was increased in LN patients compared with that in SLE patients (Figure 9A). Spatial analysis showed distinct localization patterns: IFN‐α was highly concentrated in renal tubules, while *MX2* was expressed in both glomeruli and tubules, exhibiting a characteristic bright perinuclear staining in the latter. Furthermore, *IRF7* was not only primarily localized in glomeruli but also showed elevated expression in the renal interstitium of LN patients (Figure 9B). These results, supported by Mean Fluorescence intensity (MFI) quantification, confirm the aberrant activation of the type I IFN pathway in the kidneys of LN patients.

## 4. Discussion

LN is a common and serious complication of SLE [16]. Many current studies are focused on investigating the impacts of IFN signaling on SLE, as increased IFN signaling is evident in the sera of SLE patients and may therefore serve as a potential indicator to identify disease and monitor disease activity [17]. Here, we observed that patients with moderate to high disease activity (especially in patients with LN) show evidence of elevated IFN signaling in the serum compared with patients with low disease activity, indicating that aberrantly activated type I IFN signaling may be a differentiating biomarker between SLE and LN and is closely related to LN‐associated inflammation [18]. The glomerular endothelium, mesangial cells, and macrophages become activated following exposure to immunostimulatory nucleic acids, triggering the release of higher concentrations of proinflammatory cytokines, IFN‐α, and IFN‐β [19]. Hyperproliferative IFN‐α and IFN‐β trigger signaling cascades that initiate the transcription of IFN‐stimulated genes by activating JAK1, TYK2, STAT1, and STAT2, resulting in immune dysregulation in SLE and LN [20].

Expression levels of IFN‐related genes (including *MX2*, *IRF7*, *TRIM69*, and *IFIT3*) were significantly higher in multiple disease target organs such as the kidney, and the expression profiles of IFN‐inducible genes were similar to those observed in the sera of patients with proliferative LN [21]. Among these genes, *MX2* is produced and upregulated by IFN stimulation, has strong antiviral activity against HIV‐1 and herpesvirus [22], and our findings show that MX2 expression is strongly correlated with neutrophil signatures in whole blood. This is consistent with previous reports suggesting that MX2 is involved in cellular processes that could influence neutrophil function, such as the NOD‐like receptor signaling pathway, a mechanism that warrants further mechanistic investigation regarding its role in the progression to LN. *MX2* may therefore be investigated as a candidate indicator associated with SLE activity [23] and can be investigated as a potential therapeutic option to reduce SLE and LN activity. As a member of the IFN regulatory factor (IRF) family, IRF7 mainly acts to regulate IFN transcription and regulate the expression of many genes associated with the IFN pathway. More importantly, IRF7 regulators are associated with increased immune responses, apoptosis, and viral latency. It can activate antiviral signaling factors such as *MX2* and *EIF2AK2* through p53 signaling activation, promoting IFN‐γ production [24]. In addition, genetic polymorphisms in *IRF7* are associated with SLE susceptibility [25, 26], and their expression is positively associated with relapsing lupus activity. IFN‐related genes, such as *TRIM69* and *IFIT3*, are involved in immune responses in SLE and are associated with disease activity. *TRIM69* is an IFN‐stimulated gene that is stimulated by type‐I IFN‐α/IFN‐β induction and depends on its own E3 ubiquitin ligase activity [27]. High expression of type I IFNs in SLE and LN patients is associated with a transcriptional site induced by DNA hypomethylation in naive CD4^+^ T cells, and IFN‐regulated genes show persistent hypomethylation, with *TRIM69* hypomethylation being a unique epigenetic signature in SLE and LN patients with a history of zygomatic erythematosus [28]. These studies suggest that SLE and LN patients demonstrate spontaneous activation of genes associated with the IFN‐α pathway after disease onset that not only exert direct antiviral effects but also maintain the balance of type I IFN signaling through positive or negative regulation. Abnormal type‐I IFN signaling leads to aberrant immune responses, which may trigger chronic inflammatory and autoimmune diseases such as LN. As such, investigations into the molecular mechanisms of these genes are of critical importance. Additionally, the expression of these genes in the peripheral blood of LN patients was consistent with that observed in our study, further highlighting the potential clinical value of our findings.

We found that the expression of several genes was associated with immune cell infiltration in SLE and LN patients. Specifically, the degree of neutrophil infiltration was positively correlated with the expression levels of IFN‐stimulated genes, such as *MX2* and *IRF7*. Neutrophils are major players in host innate immune responses and initiate cell death mechanisms such as NETs [29]. Neutrophils induce autoimmunity and autoantibody production in all stages of SLE by releasing NETs and play a role in endothelial injury, thrombosis, and vascular injury in diseased skin [30], which in turn activate plasmacytoid dendritic cells to secrete type I IFNs [31] and other proinflammatory factors and promote the sustained activation of SLE immune responses. Studies have shown that LN patients with high IFN activity have higher NET release levels than those with low IFN, suggesting that increased NET release or decreased degradation is associated with exacerbated autoimmunity and inflammation in LN, so inhibition of NET release may benefit LN patients [32]. In addition to neutrophils, LN patients have defects in B cell regulation that lead to their hyperactivation, differentiation, and induction of autoantibody and cytokine release [33], including type I IFN‐related cytokines such as IFN‐α. Memory B cell expression is observed to be high in the blood of SLE patients [34] and is associated with disease activity and the presence of antibodies targeting double‐stranded DNA. Switching memory B cells (CD19^+^CD27^+^IgD^–^) and double‐negative B cells (CD19^+^CD27^–^IgD^–^) were both higher in LN patients [35]. Type I IFNs affect B cell function through various mechanisms; for example, BAFF expression is upregulated by type I IFNs induced by IFN regulators (IRF1 and IRF2) [36]. Thus, therapies targeting B cells may suppress type‐I IFN activation and suppress immune responses. Together, these findings suggest that changes in the expression of genes involved in the type‐I IFN pathway may play a critical role in the immune response in LN patients, affecting immune cell infiltration and therefore patient prognosis.

In our latest study, we investigated the clinical features associated with the IFN signaling pathway, and we found that IFN‐related genes (including *MX2* and *IRF7*) and IFN‐α were highly expressed in LN patients, which was significantly different from that observed in HC and SLE patients and was closely related to the disease activity. Levels of UTP at 24 h were positively correlated with *MX2* and *IRF7* expression and could be used as an evaluation index for disease progression in SLE and LN. This may be related to the abnormal activation of type‐I IFN pathway signaling in LN pathogenesis, whose activity increases with aggravated LN inflammation. Using IF, we observed that IFN‐α was more highly expressed in tubules, *IRF7* was mostly expressed in glomeruli, and *MX2* was expressed in both glomeruli and tubules. Therefore, combining assessment of gene expression levels with existing diagnostic indicators can potentially enhance noninvasive risk assessment strategies.

We sought blood‐based biomarkers for diagnosis and prognosis in SLE. Our data indicate that type‐I IFN signaling is strongly associated with systemic disease activity and, in our biopsyanchored validation cohort, with renal involvement. These observations are associative rather than causal and require mechanistic validation. Several key limitations must be acknowledged. First, our clinical validation was conducted in a single center with a relatively small sample size (*n* = 40), and the cross‐sectional design precludes establishing causality or predictive utility for future renal flares. Second, while our cohort was rigorously selected to be treatment‐naïve to minimize medication confounding, external replication in larger, multicenter prospective cohorts is essential. Third, the protein‐level validation via immunoblotting relied on a small subset of samples (*n* = 3 per group), serving as illustrative support rather than robust quantitative confirmation. Finally, incomplete LN clinical annotation in the public datasets limited our capacity for deeper correlative subanalyses.

## 5. Conclusion

**Figure 10:**
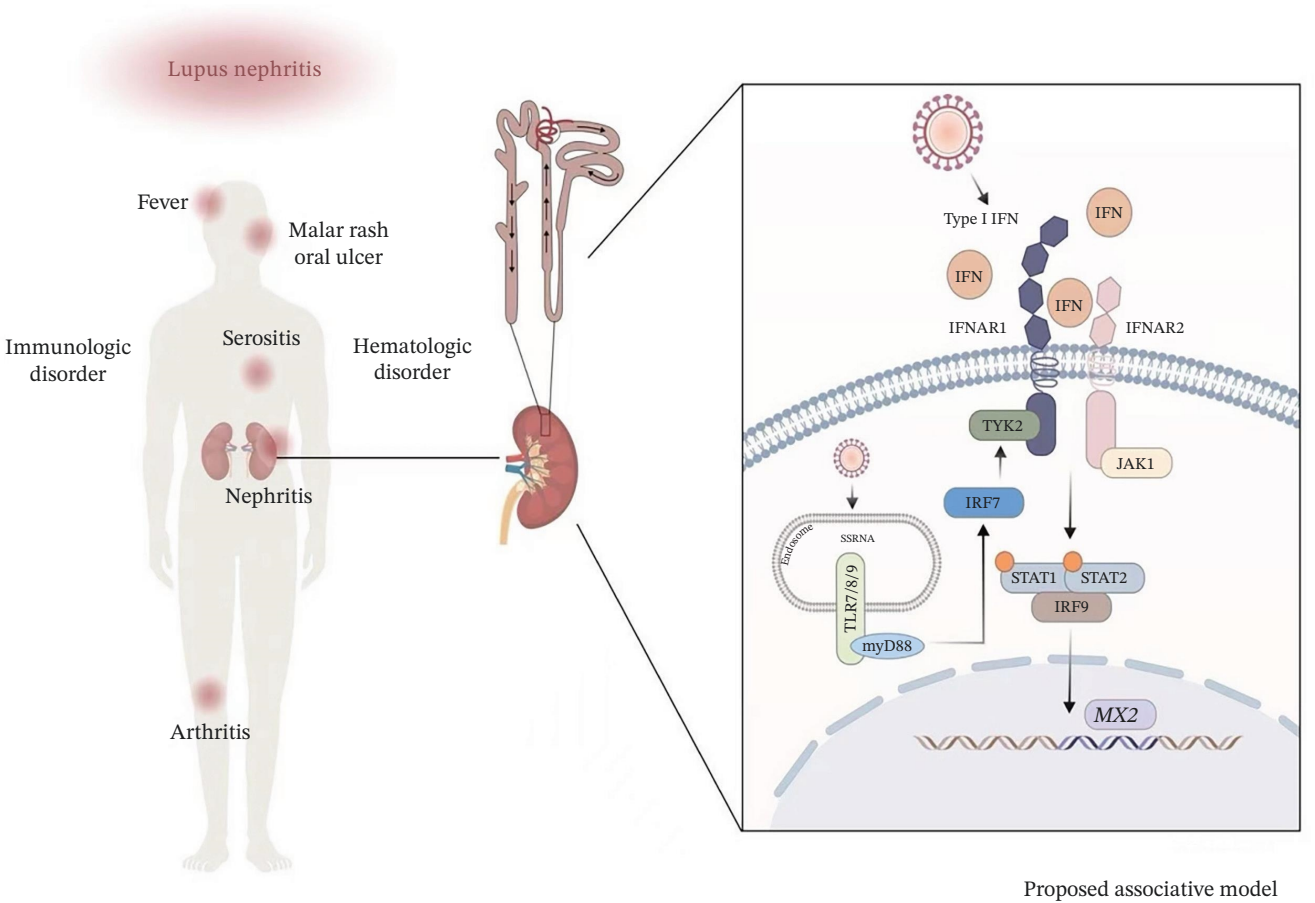
Proposed schematic model illustrating the association of type I IFN signaling with LN pathogenesis.

We identify type I IFN signaling as a blood‐based associative marker of disease activity in SLE and as preliminarily informative for renal involvement (Figure 10). Integrating ELISA, real‐time quantitative PCR results, and clinical data, MX2 and IRF7 emerged as candidate indicators associated with LN. However, given the exploratory and cross‐sectional nature of this study, these findings remain associative rather than predictive. Prospective, longitudinal studies in independent cohorts are strictly required to validate the clinical utility of these candidate markers, establish diagnostic cut‐offs, and further elucidate their mechanistic roles in LN pathogenesis.

## Author Contributions

FengQi Zhang, YiChen Huang, and Hang Liu were responsible for research design, cell experiments, data analyses, figure preparation, and writing manuscript. YiYang Zhang and ZhiYu Li contributed to clinical sample collection and data curation. ZhiYu Li and ZhiJun Xie contributed to cell experiments. Jing Sun contributed to research design, methodology, and writing reviewing and editing this paper.

## Funding

The present study was financially supported by the Zhejiang Key R&D Program (Grant 2024C03191), the National Natural Science Foundation of China (Grant 82374395), the National Natural Science Foundation of China (Grant 82405084), the Zhejiang Provincial Science and Technology Program of Traditional Chinese Medicine (Grant 2024ZR016), and the 2024 Zhejiang Chinese Medicine University Cultivation Plan for Top Innovative Talents of Postgraduates (Grant 2024YJSBJ005).

## Disclosure

All authors read and approved the manuscript.

## Ethics Statement

Approval for the current study was granted by the Ethics Committee of The Second Affiliated Hospital of Zhejiang Chinese Medical University (2022YAN No. 086‐01).

## Consent

The authors have nothing to report.

## Conflicts of Interest

The authors declare no conflicts of interest.

## Supporting Information

Additional supporting information can be found online in the Supporting Information section.

## Supporting information


**Supporting Information** Figure S1: Illustrates the flow diagram of patient recruitment and group allocation for the validation cohort.

## Data Availability

Data are available upon request from the authors.
