# Supplementary figures and images for: MX2 and IRF7 are Associated With Disease Activity and Renal Involvement in Systemic Lupus Erythematosus: An Exploratory Study
Source: J Immunol Res. 2026 Jul 26;2026:8603997. doi: 10.1155/jimr/8603997 (PMC13402894; doi:10.1155/jimr/8603997)

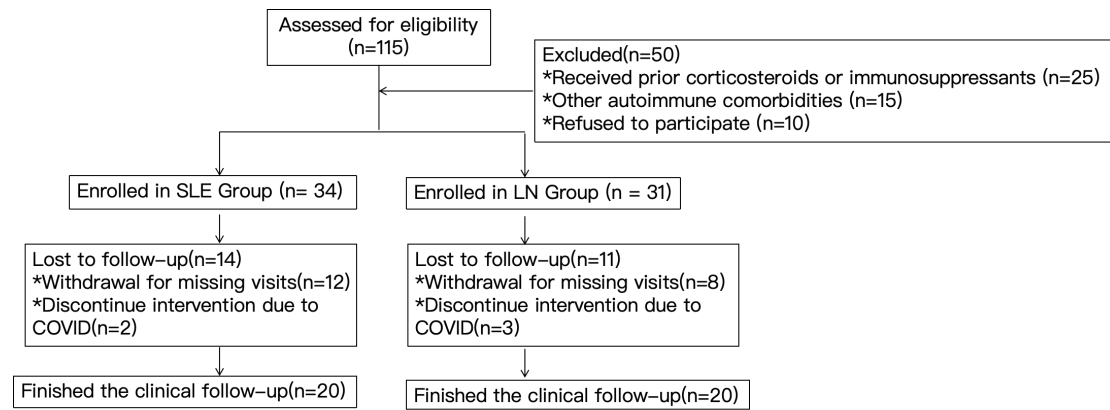

S1.Flowchart of Participant Progression in a Clinical Study

Supplement: Supplementary file 1 — Supporting Information Figure S1: Illustrates the flow diagram of patient recruitment and group allocation for the validation cohort. [file JIMR-2026-8603997-s001.pdf]
